# Synthesis of novel MgO-ZnO nanocomposite using *Pluchea indica* leaf extract and study of their biological activities

**DOI:** 10.1186/s40643-025-00848-x

**Published:** 2025-04-12

**Authors:** Samy Selim, Mohammed S. Almuhayawi, Amna A. Saddiq, Mohammed H. Alruhaili, Ebrahim Saied, Mohamed H. Sharaf, Muyassar K. Tarabulsi, Soad K. Al Jaouni

**Affiliations:** 1 Department of Clinical Laboratory Sciences, College of Applied Medical Sciences, Jouf University, Sakaka, Saudi Arabia; 2 Department of Clinical Microbiology and Immunology, Faculty of Medicine, King Abdulaziz University, 21589 Jeddah, Saudi Arabia; 3 Department of Biological Sciences, Faculty of Science, University of Jeddah, Jeddah, Saudi Arabia; 4 Special Infectious Agents Unit, King Fahad Medical Research Center, King AbdulAziz University, Jeddah, Saudi Arabia; 5 Botany and Microbiology Department, Faculty of Science, Al-Azhar University, P.O. Box 11884, Cairo, Egypt; 6 Department of Basic Medical Sciences, College of Medicine, University of Jeddah, Jeddah, Saudi Arabia; 7 Department of Hematology/Oncology, Faculty of Medicine, Yousef Abdulatif Jameel Scientific Chair of Prophetic Medicine Application, King Abdulaziz University, 21589 Jeddah, Saudi Arabia

**Keywords:** Bimetallic nanoparticles, *Pluchea indica*, Cytotoxicity, Antioxidant activity, Antibacterial

## Abstract

The biosynthesis of bimetallic nanoparticles using plant extracts has garnered significant attention due to their eco-friendly and cost-effective nature. This study aimed to biosynthesize magnesium oxide-zinc oxide nanocomposite (MgO-ZnO nanocomposite) using *Pluchea indica* leaf extract for the first time, with a focus on characterizing its physicochemical properties and evaluating its biological activities. The biosynthesized MgO-ZnO nanocomposite was fully characterized, revealing an absorbance peak at 300 nm using UV–vis spectroscopy. Transmission electron microscopy (TEM) confirmed particle stability within the size range of 5–35 nm. Cytotoxicity analysis on the Wi 38 normal cell line demonstrated an IC_50_ value of 179.13 µg/mL, indicating good biosafety. The nanocomposite exhibited potent anticancer activity, with IC_50_ values of 73.61 µg/mL and 31.25 µg/mL against Hep-G2 and MCF-7 cancer cell lines, respectively. Antibacterial assays revealed activity against *Klebsiella pneumoniae, Escherichia coli, Bacillus cereus, Staphylococcus aureus*, and *Candida albicans*, with minimum inhibitory concentrations (MICs) ranging from 31.25 to 250 µg/mL. Furthermore, the nanocomposite displayed antioxidant activity with an IC_50_ value of 175 µg/mL, as determined by the DPPH assay. In conclusion, the successful synthesis of the MgO-ZnO nanocomposite using *P. indica* leaf extract demonstrates its potential as a safe and effective agent for concentration-dependent antioxidants, antibacterial, and anticancer applications. This study highlights the versatility of plant-mediated biosynthesis in developing functional nanomaterials for biomedical use.

**Graphical Abstract:**

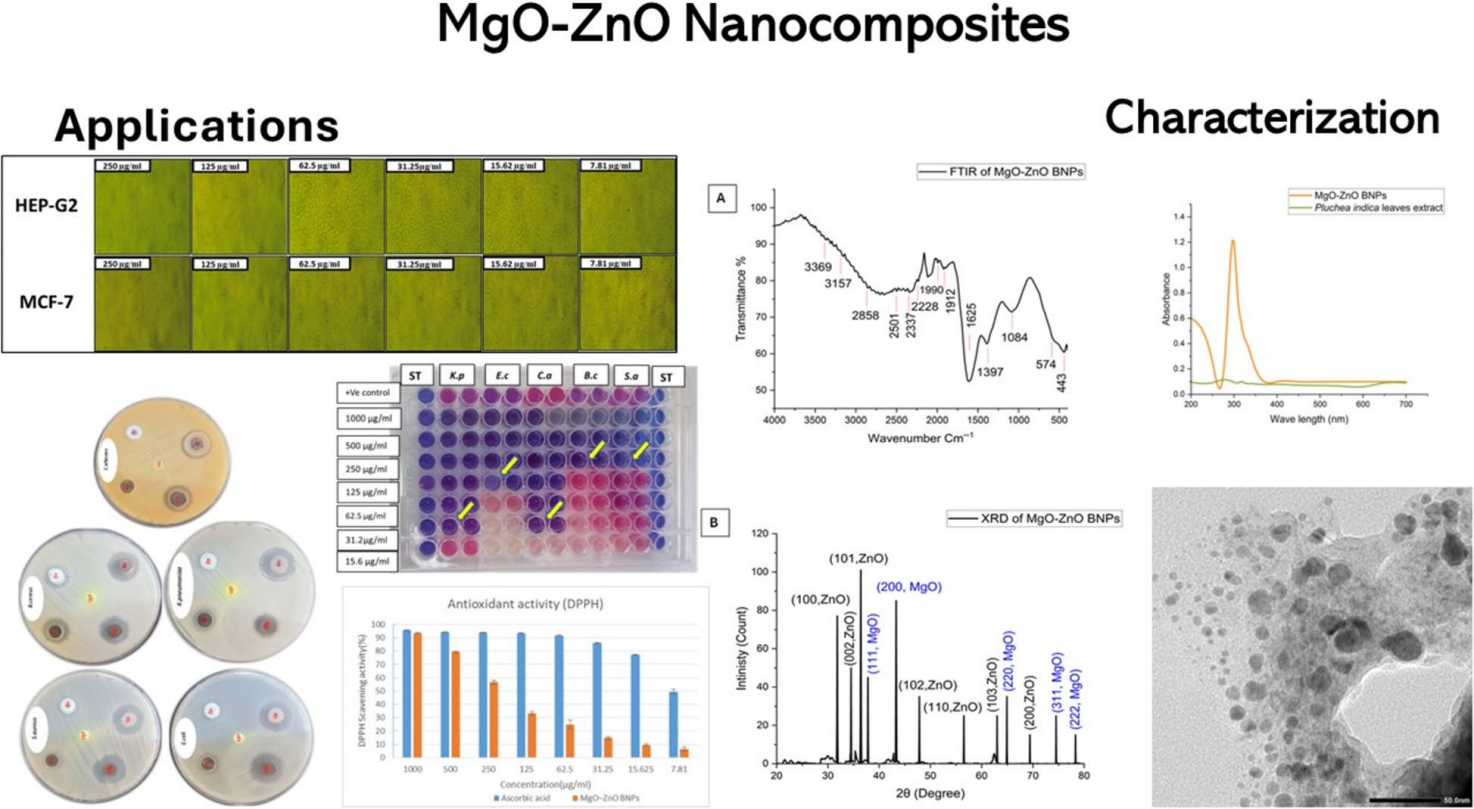

## Introduction

Bionanotechnology is the convergence of biology and nanotechnology, emphasizing the design and application of nanoscale materials and devices for biological and medical purposes (Ali et al. 2024; Saied et al. 2022; Hashem and El-Sayyad 2023). There are several ways to create nanomaterials, including chemical, physical, and biocompatible techniques. However, many chemical and physical processes generate substantial amounts of hazardous byproducts, creating challenges for waste management and potentially polluting the environment (Hasan et al. 2018). The green synthesis of nanoparticles has garnered significant attention in recent years due to its capacity to produce safe nanoparticles in a cost-effective and environmentally sustainable manner (Samuel et al. 2022). Plant parts are the best biological sources because they provide a more favorable platform for the environmentally friendly synthesis of nanoparticles, are more affordable, don't contain any hazardous chemicals, react more quickly, and produce more stable nanoparticles than other biological sources (Mohammadinejad et al. 2016; Das et al. 2017). Secondary and primary metabolites, including proteins, vitamins, enzymes, phenolics, terpenoids, alkaloids, saponins, tannins, and quinones, are used in green synthesis techniques. These biomolecules play a crucial role in stabilizing metal ions during the production process and reducing them from their precursor salts (Godeto et al. 2023). Furthermore, they facilitate the capping and surface modification of nanoparticles produced through green synthesis, which plays a crucial role in preventing the aggregation of these nanoparticles (Ali et al. 2016). Recent research has demonstrated that leaf extracts from various plant species, such as *Cynodon dactylon, Cyperus rotundus* (Suresh et al. 2020), *Moringa oleifera* (Shousha et al. 2019), *Simarouba glauca* (Thangamani and Bhuvaneshwari 2019), and *Solanum trilobatum* (Ezhilarasi et al. 2020), have been shown in recent research to be particularly successful in the synthesis of metal oxide semiconductor nanoparticles. *P. indica* leaf extract was used in this study for several reasons. This plant extract is rich in bioactive compounds like phenols, flavonoids, and saponins, which are key to facilitating the green synthesis of nanoparticles (Soni et al. 2021). These compounds help reduce metal ions and stabilize the nanoparticles, giving them unique properties. The sustainability of this method is particularly appealing since it offers a safe and eco-friendly alternative to the traditional chemical-based approaches (Saraswat et al. 2023). Other than being an eco-friendly process, the nanoparticles synthesized from *P. indica* have demonstrated the presence of prominent antioxidant, antibacterial, and antifungal activities, thus proving quite versatile in medical treatments, protection of the environment, and even food safety (Tanwar et al. 2024). Using such a plant extract not only do we gain from the natural benefits that come along with its usage but also venture toward more safe and innovative solutions in the field of industries (Tabassum et al. 2023). The biological activity of nanoparticles produced by green synthesis can be influenced by a range of biomolecules, including proteins, nucleic acids, membrane lipids, and enzymes. Formulations containing zinc oxide nanoparticles have proven effective in both medicinal and cosmetic applications (Cheraghipour et al. 2023a; Mirzaei et al. 2021a). Superior optical, semiconductivity, and piezoelectric qualities make ZnO-NPs valuable in a variety of industries, such as electronics, textiles, health, energy storage, cosmetics, and catalysis. ZnO-NPs also have antibacterial, antifungal, antioxidant, anticancer, and antiparasitic qualities, among other biomedical uses (Cheraghipour et al. 2023b). The antibacterial characteristics of zinc oxide render it a widely utilized component in topical formulations aimed at addressing blisters, dry skin, nappy rash, and various forms of skin irritation. Examples of such products include powders, antiseptic creams, surgical tapes, and shampoos. Moreover, zinc oxide is mixed with eugenol to make zinc oxide eugenol, a substance widely used in dentistry, and with iron oxide to make calamine lotion (Akbar et al. 2020; Joghee et al. 2019). On another study, zinc oxide nanoparticles-coated with eugenol (ZnO@Eug) were synthesized and evaluated against Toxoplasma gondii in vitro and in vivo (Cheraghipour et al. 2023b).

Magnesium oxide is another versatile material widely used across various industries, including catalysis, paints, refractory materials, and superconductors (Fahmy et al. 2022). Plant extracts have been used widely for the biosynthesis of magnesium oxide nanoparticles (Nguyen et al. 2023). Plant extracts such as *Clitoria ternatea*, neem leaves (Rahman 2021), Brassica oleracea, pomegranate peels (*Punica granatum*) (Sugirtha et al. 2014), and *Nephelium lappaceum* L. peel extracts (Saidi et al. 2020) have been used in several experiments to synthesize MgO NPs.

This study aimed, for the first time, to biosynthesize an MgO-ZnO nanocomposite using *P. indica* leaf extract. The biosynthesized nanocomposite was characterized using commonly-used techniques, and its biosafety, anticancer, antimicrobial, and antioxidant activities were thoroughly evaluated.

## Materials and methods

Analytical-grade magnesium nitrate (99.9%) and zinc acetate dihydrate (98.9%) were procured and used without further purification.

### Preparation of *P. indica* leaf extract

The leaf of P. indica was obtained from the Giza Governorate in Egypt. To remove any potential dirt or contaminants, the leaves were carefully cleaned using double-distilled water. The samples were permitted to equilibrate to ambient temperature through exposure to air. 200 mL of double-distilled water were combined with 10 g of finely crushed dry leaves in a 250 mL round-bottom flask. The mixture was subjected to a 60-min reflux process. The extract was filtered using Whatman filter paper No. 1 after it had reached room temperature (Al-Askar et al. 2023).

### Phytogenic synthesis of MgO-ZnO nanocomposite

The precursors for MgO-ZnO nanocomposite were created by combining equal volumes of 1 mM each of Zn(CH_3_COO)_2_·2H_2_O and Mg(NO_3_)_2_·6H_2_O. Subsequently, 120 mL of the precursor mixture and 20 mL of the extract were reacted for one hour at room temperature in a static environment. The noticeable change in the hue of the experimental solutions, transitioning from yellow to pale brown, signifies the effective production of MgO-ZnO metal nanoparticles. The light brown slurry obtained was centrifuged for five minutes at approximately 15,000 revolutions per minute (rpm) to separate the precipitate formed during the process. The solid material was thoroughly washed several times with ethanol and deionized water before being dried for 12 h at a temperature of 120 °C (Kaabo et al. 2024).

### Instruments and chemical features of NPs

A UV–visible absorption spectrophotometer (JENWAY 6305, Staffordshire, UK) was used to test the optical characteristics of the produced bimetallic nanoparticles. The observations were made throughout a wavelength range of 200–700 nm. The functional groups in charge of the stability and biogenesis of the nanoparticles were found using FT-IR spectroscopy. This experiment was conducted using a Cary 660 model and the KBr pellet method over a wavenumber range of 400–4000 cm⁻^1^. The dimensions, morphology, clustering, and crystalline structure of the nanoparticles were analyzed using transmission electron microscopy, conducted with a JEM-2100 Plus instrument (Jeol, Japan) on a carbon-coated grid. Employing an operating voltage of 40 kV and a current of 30 mA, along with a 2θ angle range spanning from 10° to 80°, a Shimadzu XRD-6000 system (Shimadzu Scientific Instruments, Japan) was utilized to conduct a detailed examination of the crystalline structure of the MgO-ZnO nanocomposite. Furthermore, dynamic light scattering (Nano ZS, Malvern, UK) was using to assess the stability and average particle size distribution of the synthesized MgO-ZnO nanocomposite.

### Cytotoxicity and anticancer activity

The Wi 38 normal cell line was used to assess the cytotoxic impact of the MgO-ZnO nanocomposite using the MTT assay (Loosdrecht et al. 1994). The anticancer activity was also investigated against the cancerous Hep-G2 and MCF-7 cell lines, which were obtained from the American Type Culture Collection (ATCC). A 96-well tissue culture plate was inoculated with a cell suspension of 1 × 10^5^ cells/mL, with 100 µL dispensed into each well. The plate was incubated at 37 °C for 24 h to facilitate the formation of a confluent monolayer of cells. Upon achieving confluency, the growth medium was carefully aspirated, and the cell monolayer was washed twice with wash media to remove any residual serum. Subsequently, a series of two-fold dilutions of the test sample was prepared in RPMI medium supplemented with 2% serum, referred to as the maintenance medium. A total of 0.1 mL of each dilution was dispensed into designated wells, while three wells were reserved as controls, receiving only the maintenance medium. The plate was then incubated at 37 °C and monitored for physical signs of cytotoxicity, including partial or complete loss of the monolayer, cellular rounding, shrinkage, or granulation. To assess cell viability, an MTT assay was performed. MTT solution was prepared at a concentration of 5 mg/mL in phosphate-buffered saline (PBS) (BIO BASIC CANADA INC). Following incubation, 20 µL of the MTT solution was added to each well, and the plate was shaken at 150 rpm for 5 min to ensure thorough mixing. The plate was then incubated at 37 °C in a 5% CO2 atmosphere for 4 h, allowing for the metabolic conversion of MTT to formazan crystals. The optical density of the samples was measured at a wavelength of approximately 560 nm. The following formula was used to determine the total cell count and the proportion of viable cells.

$${\text{Viability \% }} = { }\frac{{\text{Test OD}}}{{\text{Control OD}}}\, \times \,100$$

$${\text{Inhibition \% }} = 100 - {\text{Viability \% }}$$

### Antimicrobial activity

The antibacterial properties of MgO-ZnO nanocomposite were assessed in this study utilizing Muller-Hinton Agar (MHA), India, for bacterial cultures and PDA for yeast cultures. standard strains of *S. aureus* (ATCC 6538), *K. pneumonia, B. cereus* (ATCC 10987), *E. coli* (ATCC 8739) in addition to *C. albicans* (ATCC10231) were cultivated on prepared MHA and PDA surfaces for bacteria and fungi, respectively, for twenty-four hours. A sterile corkborer was used to cut wells measuring 6 mm. 100 µl of each compound was then placed into each well individually and allowed to incubate for 2 h at a temperature of 4℃. For the bacterial strain, 25 µg of Co-Trimoxazole was used as the control, and for *C. albicans*, 25 µg of fluconazole was used. The plates were subsequently incubated for 24 h at 37 °C for bacteria and 48 h at 28 °C for *C. albicans*. The inhibitory zones' dimensions were measured and recorded after incubation (Sharaf et al. 2021).

### Minimum inhibitory concentraton

Following adjustments and confirmation using the broth microdilution test, El-Didamony et al*.* (2024) evaluated the MIC of MgO-ZnO nanocomposite against *S. aureus, B. cereus, E. coli, K. pneumoniae*, and *C. albicans*. To determine the MIC, different concentrations of the MgO-ZnO nanocomposite were tested, with 100 µl of each concentration added to the wells of a microtiter plate. To confirm that the bacterial suspension and MH broth were sufficient, wells designated as positive controls were filled with MH broth and a bacterial suspension. The plates were then incubated at 37 °C for 24 h. Afterward, 30 µl of HiMedia's resazurin solution (0.02% weight/volume) was added to each well, and the plate was incubated for an additional 24 h to detect any bacterial proliferation (El-Sherbiny et al. 2022).

### DPPH assay

With a few minor adjustments, the DPPH (2,2-diphenyl-1-picrylhydrazyl) method, as described by El-Sayed et al. (2023), was used to measure the antioxidant activity of the MgO-ZnO nanocomposite.

### Statistical analysis

GraphPad Prism 8.0 (CA, USA) was used for data analysis and graphical representation. All findings are presented as means ± standard deviation (SD), and all experiments were conducted in triplicate (n = 3). The statistical analysis was accomplished using one-way analysis of variance (ANOVA) and Tukey's several comparison tests, where P < 0.05 was considered significant.

## Results and discussion

### Biosynthesis of MgO-ZnO nanocomposite

The current study demonstrates the successful synthesis of MgO-ZnO nanocomposite using *P. indica* leaf extracts. The process is rapid, simple, environmentally friendly, and cost-effective. Upon mixing the leaf extract with metal precursors, a noticeable color change from yellow to light brown occurred, indicating the formation of the MgO-ZnO nanocomposite. This transformation is attributed to the activation of surface plasmon resonance, signifying the reduction and stabilization of metal ions by the phytochemicals present in the extract (Loiseau et al. 2019). The use of P. indica extracts effectively minimized the negative impacts of conventional chemical and physical synthesis methods by acting as reducing, capping, and stabilizing agents (Badru et al. 2023).

Plants, often referred to as nature's chemical factories due to their affordability and low maintenance, are rich sources of phytochemicals such as polyols, terpenoids, and polyphenols (Ovais et al. 2018). These compounds are crucial for the bioreduction of metal ions, which is essential in green synthesis (Hano and Abbasi 2021). The findings of this study align with previous research. For instance, Sheema et al. (2024) biofabricated MgO:ZnO nanocomposites using *Malvastrum coromandelianum*, while Hashem et al. (Hashem et al. 2023a) utilized watermelon peel to biosynthesize bimetallic selenium-silver nanoparticles. In another study, ZnO@SeO nanoparticles synthesized using pomegranate peel extracts exhibited notable antibacterial, antifungal, and anticancer properties (Hashem et al. 2023b). Similarly, Venkatesan et al. (Venkatesan et al. 2024) synthesized ZnO nanoparticles with *Vitex negundo* leaf extract, Khan et al. (Khan et al. 2020) used *Camellia sinensis* leaves for MgO nanoparticles, and Sharmila et al. (Sharmila et al. 2019) employed Pisonia alba leaf extract for MgO nanoparticle synthesis. Al-Askar et al. (Al-Askar et al. 2023) investigated the antibacterial and photocatalytic properties of ZnO NPs biosynthesized using *P. indica*. Shakib et al. successfully prepared ZnO NPs using *Okra mucilage* as a green synthesis agent and evaluated their biological activities, highlighting the potential of this eco-friendly approach (Shakib et al. 2023). Mirzaei et al. (2021b) utilized *Gracilaria corticata* seaweed extract for the green synthesis of ZnSe NPs, highlighting a sustainable approach with potential biological applications. On another study, ZnSe NPs were fabricated from an aqueous extract of *Rosmarinus officinalis* L. and study their therapeutic purposes (Somaghian et al. 2024). These studies collectively underscore the versatility and potential of plant-mediated nanoparticle synthesis.

### Characterization

#### UV–vis spectroscopy

**Fig. 1:**
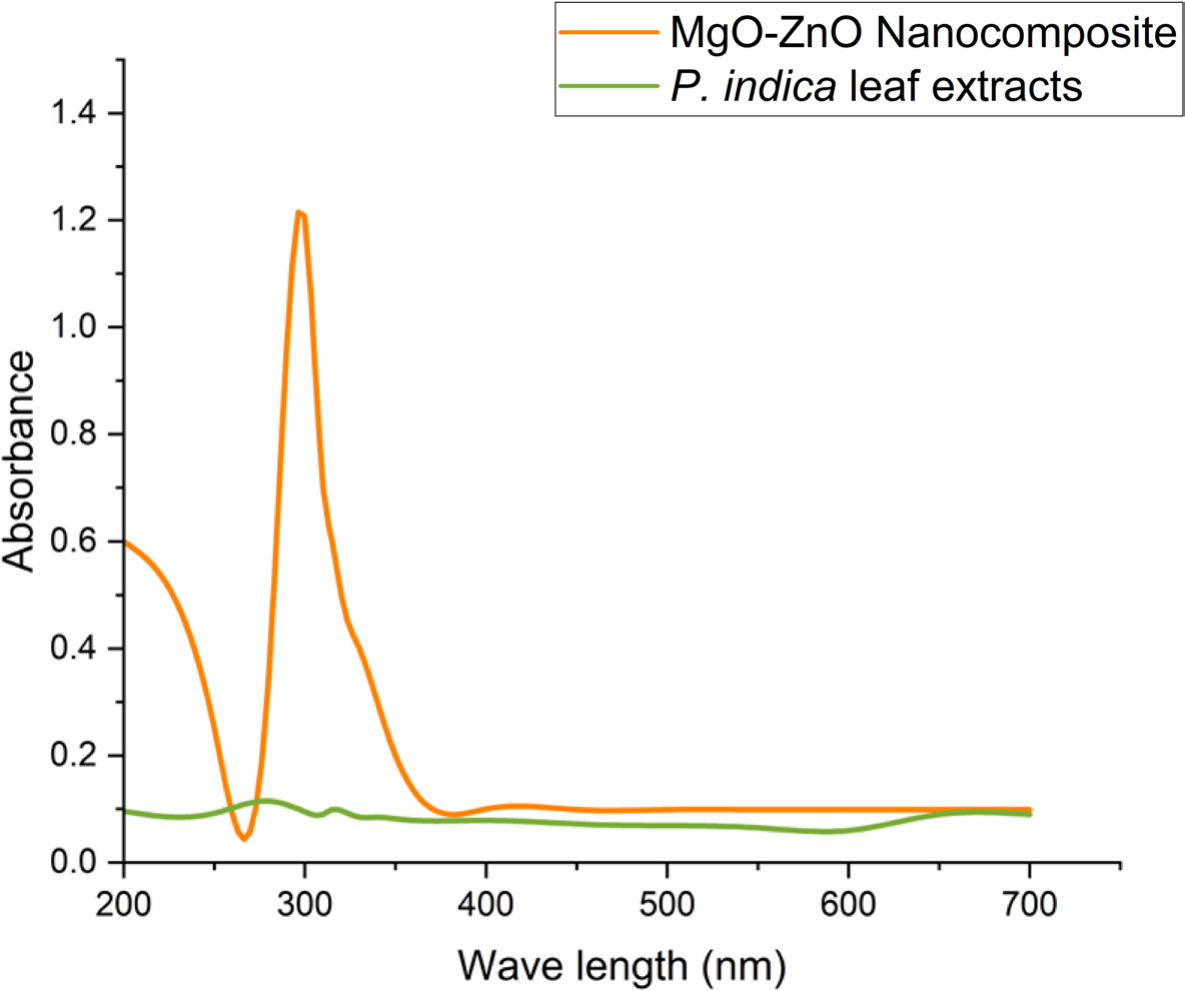
UV–Vis. spectroscopy of MgO-ZnO nanocomposite

The optical absorption properties of the BNPs were examined using a UV–vis diffuse reflectance spectrophotometer. The absorbance of the nanoparticles was measured between 200 and 700 nm to determine their maximal SPR. As shown in Fig. 1, the biosynthesized MgO-ZnO nanocomposite's greatest SPR was seen at 300 nm. Similarly, according to Somaghian et al. (2024) the UV–Vis spectrum, a distinct peak at 398 nm corresponds to the formation of ZnSe NPs by the *R. officinalis* L extract. Mohammed et al. (2022) revealed that the SPR ranges for CuO/ZnO core/shell nanoparticles and MgO/ZnO core/shell nanoparticles were, respectively, 300–400 and 300–500 nm. The nanoparticles were detected by UV–Vis examination at 370 nm of bimetallic B_2_-O_3_-ZnO nanoparticles in a solution had a deep off-white color development in different research (Hashem et al. 2023c). Mirzaei et al. (2021b) revealed two absorption peaks in the 250 nm and 360 nm regions associated with the formation of ZnSe NPs. The size and form of the nanoparticles, the surrounding medium, the SPR frequency, and the metal's dielectric constant all affect the SPR wavelength (Essien et al. 2020). Peak absorbance values were found to be between 320 and 380 nm in previous studies on the green production of ZnO nanoparticles (Al-Askar et al. 2023; Fouda et al. 2023).

By using *P. indica* leaf extracts, Al-Askar et al. (2023) found that the maximum SPR for biosynthesized ZnONPs was 360 nm. after a day. UV–Vis spectroscopy was employed to confirm the phytosynthesis of magnesium oxide nanoparticles using *P. marsupium* extract, with the absorption peak observed at 310 nm (Ammulu et al. 2021). Essien et al. (2020) used the MgONPs solution's UV–Vis absorption spectra to demonstrate a distinct absorption band at 260 nm. The UV–Vis spectra of the generated ZnO nanoparticles exhibited a distinct peak at 323 nm, which was consistent with the findings of Vaseem et al. (Zaater et al. 2024).

#### Fourier transform infrared spectroscopy

**Fig. 2:**
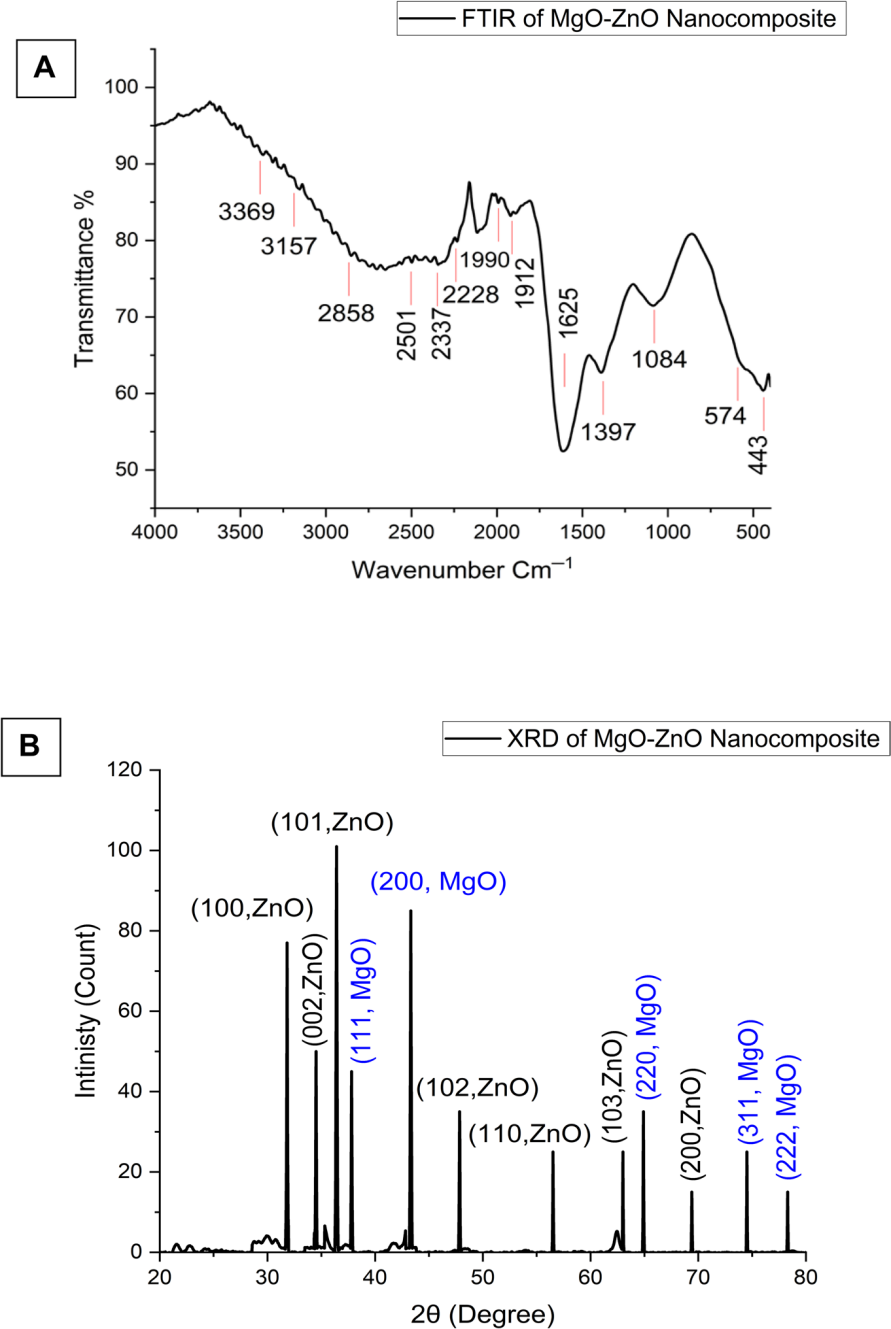
**A** FTIR of MgO-ZnO nanocomposite synthesized by using *P. indica* leaf extracts; and **B** XRD of MgO-ZnO nanocomposite

FTIR analysis was utilized to identify the various functional groups found in the *P. indica* leaf extract and to understand how they contributed to the formation and stability of the MgO-ZnO nanocomposite. Figure 2A of the FITR spectra showed the typical peaks of the MgO-ZnO nanocomposite at 3369, 3157, 2858, 2501, 2337, 2228, 1990, 1912, 1625.1397, 1084, 574, and 443 cm^−1^. Peaks at 3369 and 3157 cm⁻^1^ were found by Mphahlele et al. (2017) to represent the stretching vibrations of the amine (–NH) and hydroxyl (OH) groups in protein amide bonds, respectively. The peaks observed at 2858 and 2501 cm⁻^1^ were attributed to the stretching of CH groups in free sugars and the aldehyde group (CHO) (Seghir et al. 2023). The carbonyl group (C = O), which indicates the presence of ketones, aldehydes, and carboxylic acids, was found at 1990 and 1912 cm⁻^1^, whereas the stretching band of the alkyne group (C≡C) was found at 2337 and 2228 cm⁻^1^. Peaks at 1084 and 1397 cm⁻^3^, respectively, indicated the existence of C-N stretching vibrations in aromatic amines and alcohol and phenolic compounds (Munir et al. 2021). Furthermore, the MgO-ZnO nanocomposite was associated with peaks at 574 and 443 cm⁻^1^. This association may have resulted from interactions between Mg–O or Zn–O bonds formed by hydroxyl groups and magnesium or zinc nanoparticles. The results verified the presence of several functional groups, such as alkanes, alkenes, aliphatic and aromatic amines, and alkyls, in the *P. indica* leaf extract. These functional groups are essential for the reduction, capping, and stability of the MgO-ZnO nanocomposite. The effective production of ZnO nanoparticles in 2023 was also validated by Al-Askar et al. (2023), with the band at 414 cm⁻^1^ providing unambiguous proof of the zinc oxide bond.

#### X-ray diffraction

MgO-ZnO nanocomposite XRD experiments are shown in Fig. 2B. The XRD patterns of the MgO-ZnO nanocomposite showed broad diffraction peaks at diffraction locations of 31.8°, 34.5°, 36.4°, 47.8°, 56.5°, 63.0°, and 69.4°. The ZnO structure was associated with these peaks, which were assigned to the Miller indices (100), (002), (101), (102), (110), (103), and (200), in that order. Zinc oxide with a hexagonal wurtzite structure is present, as confirmed by XRD investigation (JCPDS card no: 36-1451). This outcome is consistent with that which Singh et al. (2023) reported; Miller indices (100), (002), (101), (102), (110), (103), and (200) corresponded to the diffraction peaks at positions 31.8°, 34.5°, 36.4°, 47.8°, 56.5°, 63.0°, and 69.4°.

Unique diffraction peaks at 2θ values of 37.8°, 43.3°, 64.9°, 74.5°, and 78.3° match the (111), (200), (220), (311), and (222) planes of a face-centered cubic (FCC) lattice structure of magnesium, suggesting that the nanoparticles of magnesium oxide are crystalline. The polycrystalline cubic structure of MgO was validated by these peaks matching JCPDS No. 89-4248. This conclusion aligns with the research conducted by Ammulu et al. (2021), who also observed significant diffraction peaks linked to the (111), (311), and (222) planes of an FCC lattice for magnesium oxide, demonstrating the crystalline nature of the substance (Essien et al. 2020; Ammulu et al. 2021). The average crystallite size was calculated using the Debye–Scherrer equation and found to be 41 nm (Sharma et al. 2017).

#### Transmission electron microscope

**Fig. 3:**
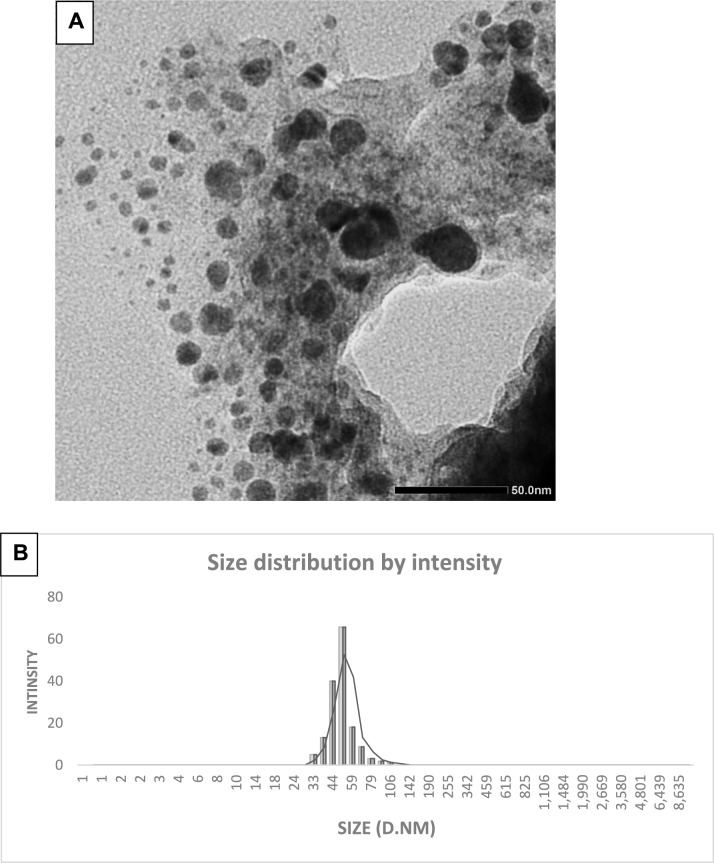
Examination of MgO-ZnO nanocomposite's **A** TEM picture and **B** DLS image

Using TEM, the average size of the biosynthesized MgO-ZnO nanocomposite was determined. The TEM picture (Fig. 3A) demonstrated the spherical forms and monodisperse nature of the MgO-ZnO nanocomposite, which ranged in size from 5 to 35 nm. These TEM results were contrasted with observations from dynamic light scattering (DLS). ZnO nanoparticles were found to be spherical in shape, with an average diameter of 20.28 nm and a size distribution ranging from 15 to 25 nm, according to the study by Dutta et al. (2023). In contrast, ZnO-Ag nanoparticles in their experiment ranged in size from 20 to 50 nm, with an average diameter of 29.77 nm. Gopinath et al. (2016) reported that bimetallic Ag/Au nanoparticles were spherical and averaged 10 nm in size. In addition, Elkady et al. (2024) measured the average particle diameter of St/Ag-Se nanoparticles, which had an oval and spherical form, confirming that the biosynthesis of the particles had been effective.

#### Dynamic light spectroscopy

Calculating the size and size distribution of nanoparticles is commonly accomplished using DLS. The analysis of light scattering resulting from Brownian motion is used to calculate the average particle size, size distribution, and polydispersity index (PDI). The study results indicated that the MgO-ZnO nanocomposite had an average particle size of 51 nm and a PDI of 0.31. For these BNPs, Fig. 3B illustrates the intensity distribution by size. A PDI value of less than 0.05 is often associated with monodisperse samples, while values higher than 0.7 indicate a broader size distribution (El-Behery et al. 2023). The findings suggest that the size distribution of the MgO-ZnO Nanocomposite is rather restricted. Because of the hydrodynamic radius of the nanoparticles and the surrounding water layers, it was noted that the particle sizes shown in TEM images were lower than the average sizes reported by DLS analysis (Nyabadza et al. 2023). In contrast, El-Behery et al. (El-Behery et al. 2023) reported that artificial bimetallic Ag-Se nanoparticles had a particle size of 66.5 nm. ZnO nanoparticles measured 131 nm, whereas ZnO–Ag nanoparticles measured 138 nm, according to Dutta et al. (2023). Furthermore, Hashem et al. (2023d) found a typical particle size distribution of 70.54 nm for bimetallic B₂O₃–ZnO nanoparticles generated by gamma radiation.

### Biosafety of MgO-ZnO nanocomposite

Evaluating the toxicity of new compounds using normal cell lines is a crucial step in assessing their biosafety. This approach involves exposing non-cancerous cell lines to various concentrations of the compounds to observe any cytotoxic effects, such as changes in cell viability, morphology, and proliferation rates (Khalil et al. 2021). By comparing the responses of normal cells to those of cancerous cells, researchers can identify compounds that specifically target cancerous cells while preserving healthy tissue. This not only helps in determining the safety profile of new drugs but also aids in understanding the mechanisms of toxicity, ultimately guiding the development of safer therapeutic agents with reduced side effects (Chehelgerdi et al. 2023).

**Fig. 4:**
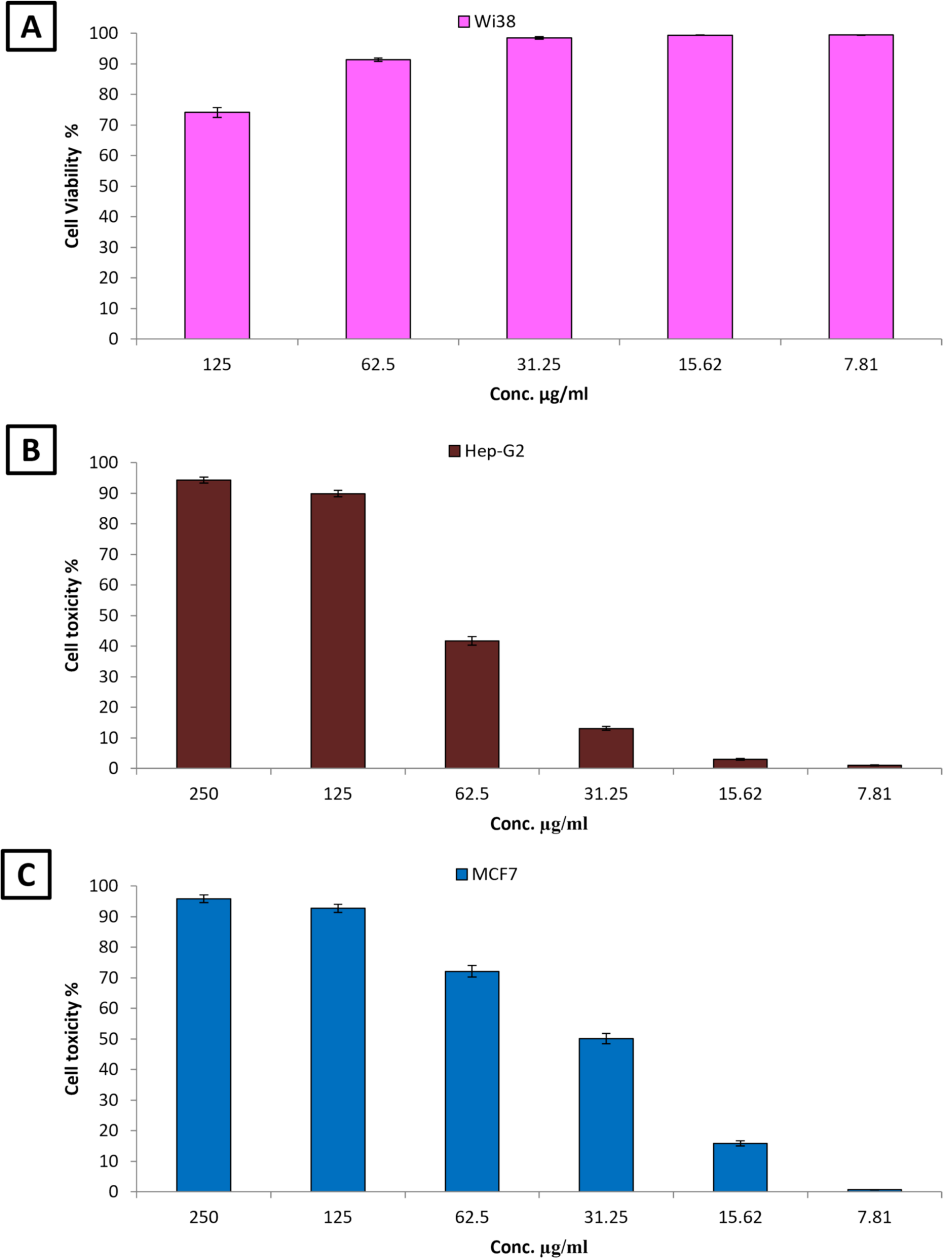
Cell viability of MgO-ZnO nanocomposite toward Wi 38 normal cell line (**A**), cell toxicity of MgO-ZnO nanocomposite against Hep-G2 (**B**) and MCF-7 (**C**) cancerous cell lines

The present investigation evaluated the toxicity of biosynthesized MgO-ZnO nanocomposite utilizing the Wi 38 normal cell line, as shown in Fig. 4A. The findings showed that the cell viability of Wi 38 normal cell line at dosages of 125, 62.5%, 31.25%, 15.62%, and 7.81 µg/mL, was 74.06, 91.35, 98.45, 99.34, and 99.44%. Moreover, the IC_50_ value of the MgO-ZnO nanocomposite against the Wi 38 cell line was determined to be 179.13 µg/mL. Generally, if the IC_50_ value is 90 μg/mL or more, the compound is classified as non-cytotoxic (Ioset et al. 2009). Therefore, the MgO–ZnO base nanoparticles produced via biosynthesis are safe for application.

### Anticancer activity of MgO-ZnO nanocomposite against Hep-G2 and MCF-7 cancerous cell lines

**Fig. 5:**
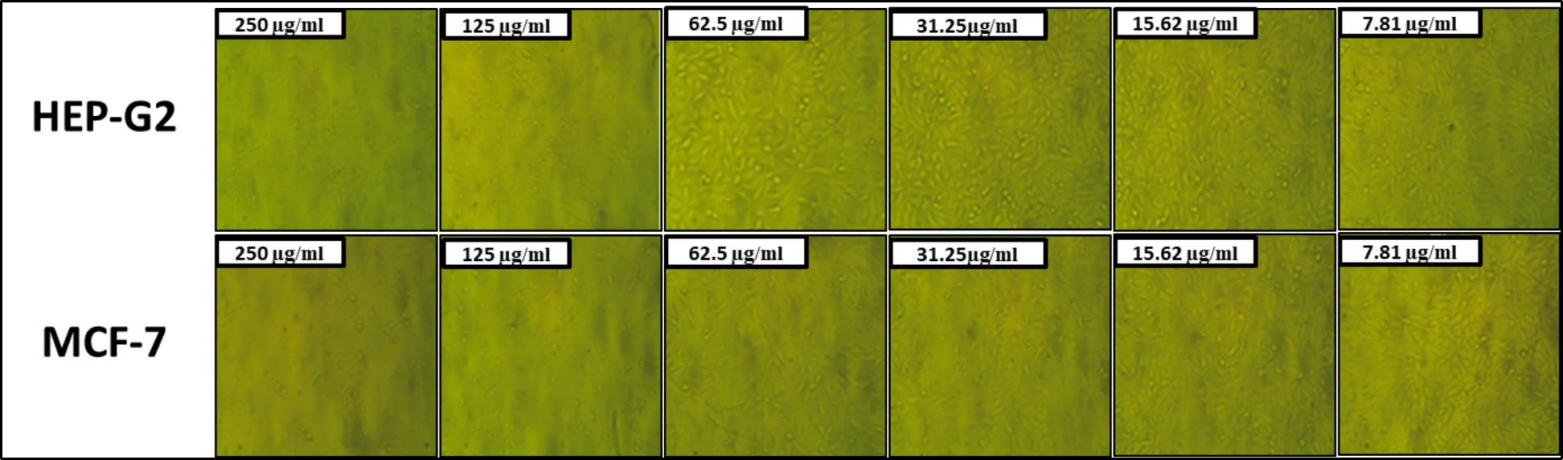
Photos of treated Hep-G2 and MCF-7 with different concentrations of MgO-ZnO nanocomposite under inverted microscope

Bimetallic nanoparticles have garnered significant attention in cancer therapy due to their unique properties that enhance anticancer activity compared to their monometallic counterparts (Niu et al. 2022). Figures 5 demonstrate the results of our evaluation of the anticancer efficacy of biosynthesized MgO-ZnO nanocomposite against the Hep-G2 and MCF-7 cancer cell lines. The biosynthesized MgO-ZnO nanocomposite exhibited strong anticancer effects against Hep-G2 and MCF-7 cells, according to the results. In addition, at doses of 250, 125, 62.5, and 31.25 µg/mL, the anticancer activity of MgO-ZnO nanocomposite against Hep-G2 was 94.26%, 89.83%, 41.71%, and 13.10%, respectively (Fig. 4B). Moreover, results showed that the IC_50_ of MgO-ZnO Nanocomposite toward Hep-G2 was 73.61 µg/mL. Likewise, MgO-ZnO nanocomposite showed excellent anticancer activity toward MCF-7, where anticancer activity at concentrations of 250, 125, 62.5, and 31.25 µg/mL was 95.77, 92.68, 72.06, and 50.0%, respectively (Fig. 4C).. Also, the IC_50_ of MgO-ZnO nanocomposite toward the MCF-7 cancerous cell line was 31.25 µg/mL.

Bimetallic nanoparticles demonstrate significant anticancer mechanisms primarily through the generation of ROS and enhanced cellular uptake. The unique properties of these nanoparticles enable them to produce ROS, which induces oxidative stress in cancer cells, leading to apoptosis via mitochondrial dysfunction and DNA damage. This mechanism is particularly effective in overcoming resistance seen in various cancers (Makada et al. 2023). Additionally, bimetallic nanoparticles can be functionalized with specific targeting ligands, allowing for selective binding to cancer cell receptors. This targeted approach enhances the delivery of therapeutic agents directly to tumor sites, thereby increasing drug accumulation and minimizing systemic toxicity (Machado et al. 2021). Moreover, bimetallic nanoparticles are utilized in photothermal therapy, where they convert light energy into heat upon irradiation, causing localized hyperthermia that selectively destroys cancer cells (Makada et al. 2023). This method can be combined with traditional treatments, such as radiotherapy, to enhance the overall therapeutic effect. The synergistic interactions between the metals in bimetallic nanoparticles can also improve their efficacy, making them more effective than their monometallic counterparts.

Prior investigations have shown that bimetallic nanoparticles display substantial anticancer properties. Using *Salvia officinalis* extract, Asghari Moghaddam et al. (2024) produced AgZnO nanoparticles and found that the combined actions of silver and zinc oxide increased the cytotoxicity against A549 lung cancer cells. Furthermore, Orshiso et al. (2023) employed an extract from *Artemisia abyssinica* leaves to produce bimetallic ZnO − CuO nanoparticles using environmentally friendly methods. These nanoparticles exhibited strong anticancer effects against the MCF-7 cell line, with an IC_50_ value of 33.12 μg/mL. Additionally, research by Hashem et al. (2023d) showed that synthesized bimetallic B_2_O_3_–ZnO nanoparticles exhibited anticancer activity against the Caco-2 cell line, with an IC_50_ of 80.1 μg/mL.

### Antimicrobial activity

**Fig. 6:**
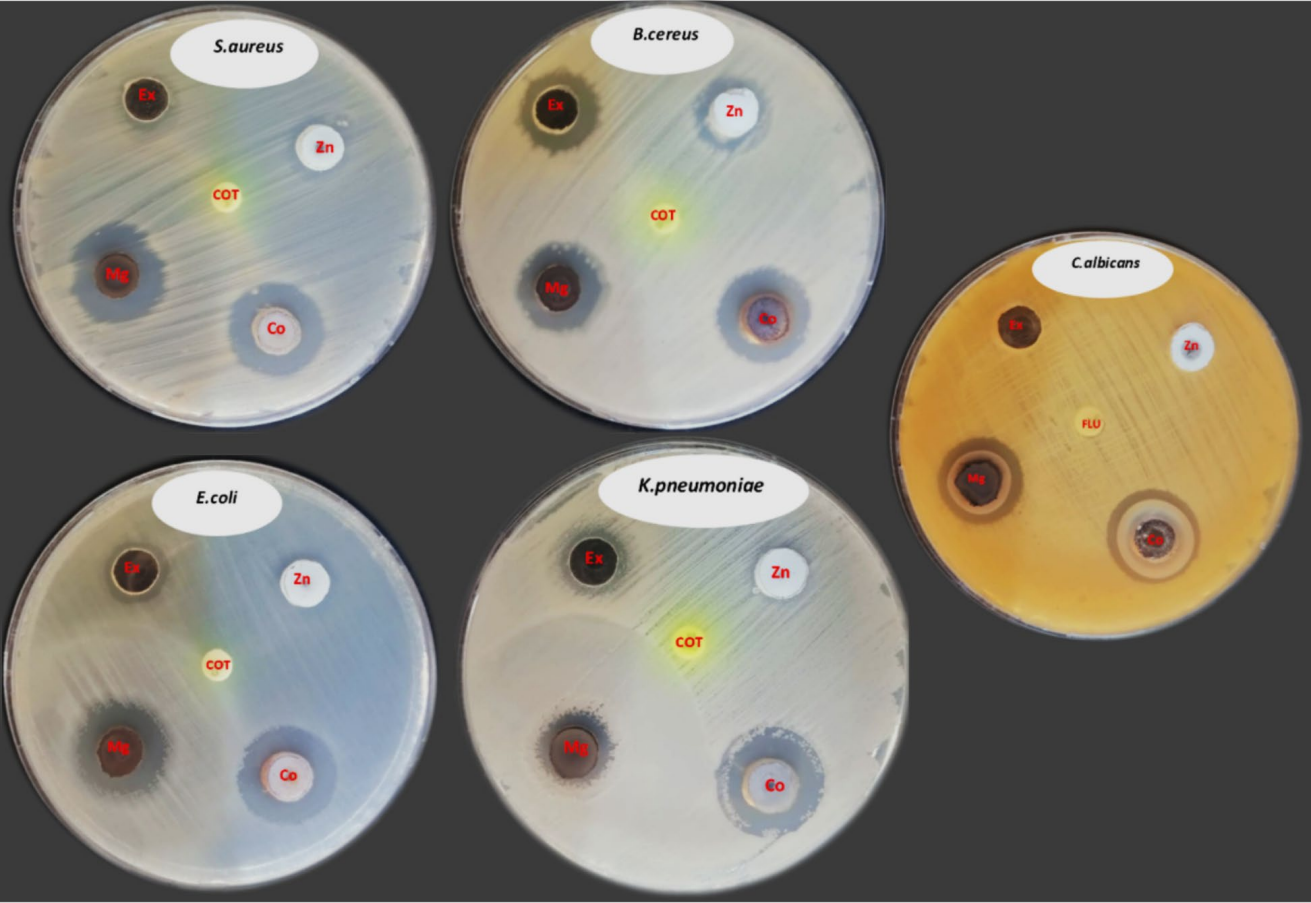
Antimicrobial activity of Zinc oxide nanoparticles (Zn), Magnesium oxide nanoparticles (Mg) and MgO-ZnO nanocomposite (Co) against (*S. aureus, B. cereus, E. coli, K. pneumonia* and *C. albicans*). Ex = Extract, COT = Co-Trimoxazole, FLU = Fluconazole

Data on the antibacterial activity of MgO and ZnO against various microbial strains, along with the effectiveness of the MgO-ZnO nanocomposite, were collected and analyzed. The MgO-ZnO nanocomposite exhibits antibacterial efficacy against both Gram-positive and Gram-negative bacteria, as well as *C. albicans*, as shown in Table 1 and Fig. 6. These particles have different sizes for their inhibitory zones. The antimicrobial activity of ZnO and MgO varies across different microbial strains, with both agents demonstrating effectiveness individually. However, their combined use always results in enhanced efficacy, highlighting the importance of understanding the interactions between different antimicrobial agents to develop effective treatment strategies. Further studies could investigate the underlying mechanisms and potential applications in clinical practice. The results indicate that ZnO and MgO have distinct antimicrobial activities. ZnO is particularly effective against *Staphylococcus aureus* and *Bacillus cereus*, while MgO shows a broader spectrum of activity, especially against *K. pneumoniae* and *E. coli*. The MgO-ZnO nanocomposite consistently enhances the antimicrobial effect, as seen with all strains, suggesting that the interaction between ZnO and MgO may be synergistic for all strains.

**Table 1:** Antimicrobial activity of ZnO, MgO and MgO-ZnO nanocomposite

Microbial strain	Inhibition zone (mm)				Co-Trimoxazole /fluconazole
	Extract	ZnO	MgO	MgO-ZnO Nanocomposite	
*Staphylococcus aureus*	11.3 ± 0.3	13.3 ± 0.3	20.3 ± 0.8	21 ± 0.5	0
*Klebsiella pneumonia*	13 ± 0.5	0	15.5 ± 0.2	21.5 ± 0.28	0
*Bacillus cereus*	16 ± 0.5	16 ± 0.5	18.6 ± 0.3	22 ± 0.5	0
*Escherichia coli*	13.6 ± 0.3	0	20 ± 0.5	21.6 ± 0.8	0
*Candida albicans*	0	0	20 ± 0.5	22 ± 0.5	0

Since ZnO NPs have little chance of harming people or animals, they are generally regarded as stable and safe antibacterial agents. These NPs have antibacterial properties because their surface releases ROS. These ROS damage the proteins, DNA, and membranes of bacteria when they come into contact with them and accumulate within the microorganisms. This process is known as oxidative stress. Moreover, these NPs cause the cells to produce antimicrobial ions (Zn^2+^), creating a toxic milieu that eventually damages the intracellular area and membrane (Seghir et al. 2023).

MgO NPs and ZnO NPs differ from one another in terms of their antibacterial properties. In general, bacteria can be killed by magnesium oxide and zinc oxide due to their antibacterial properties (Naikoo et al. 2021; Iravani 2011). The capacity of zinc oxide to emit harmful zinc ions to bacteria is the reason behind this. These ions can interfere with bacterial cell membranes, alter biological functions, and result in cell death. However, we suggest many processes by which MgO nanoparticles affect bacteria: Increased concentrations of nanoparticles result in significant damage to the cell membrane, loss of cell contents, and physical contact between the nanoparticles and the cell surface, which destroys the integrity of the bacterial membrane and permits membrane leakage. (Hajam et al. 2020) When suspended, MgO nanoparticles consistently produce a specific amount of H_2_O_2_, which causes oxidative stress in cells. (2) Elevated levels of nanoparticles lead to significant impairment of the cell membrane, the liberation of cellular contents, irreversible oxidation of biomolecules (such as DNA, proteins, and lipids), and finally destruction of the cell. (3) Nanoparticles' physical contact with the cell surface compromises the integrity of bacterial membranes and leads to membrane leakage (He et al. 2016).

### Determination of MIC

**Fig. 7:**
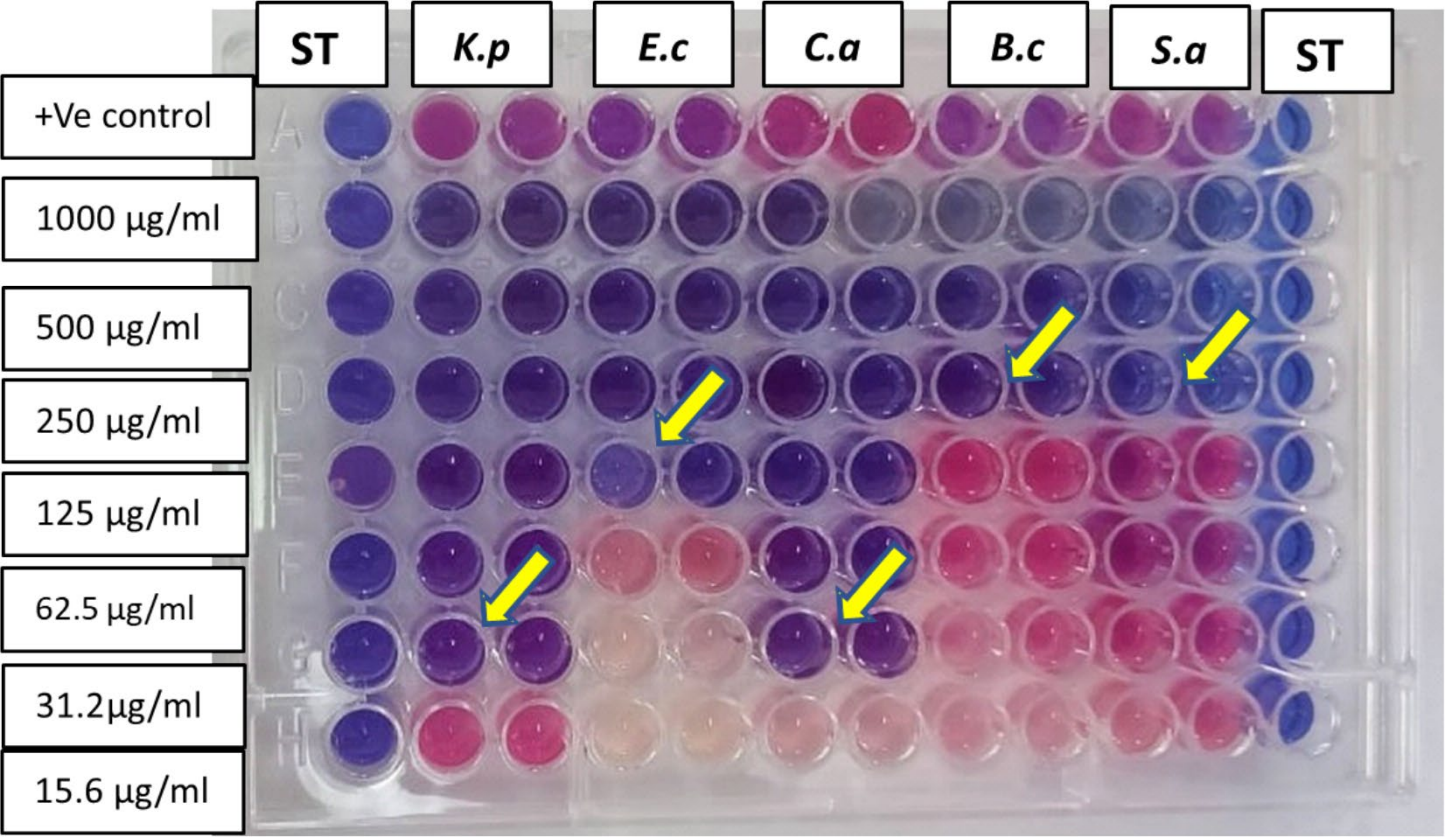
MIC of MgO-ZnO nanocomposite on (Ec = *E. coli*),(KP = *K. pneumonia*),(Sa = *S. aureus*),(Bc = *B. cereus*) and (Ca = *C. albicans*)

The MIC of MgO-ZnO nanocomposite against several bacterial strains and the fungus *C. albicans* is displayed in Table 2. As shown in Table 2 and Fig. 7, the minimum concentration of nanoparticles required to inhibit the growth of these microbes was indicated by the MIC values, which ranged from 31.2 to 250 μg/mL.

**Table 2:** MIC of MgO–ZnO BNPs against bacterial strains and *C. albicans*

Microbial strain	MIC of MgO-ZnO nanocomposite (µg/mL)
*Staphylococcus aureus*	250
*Bacillus cereus*	250
*Klebsiella pneumonia*	31.2
*Escherichia coli*	125
*Candida albicans*	31.2

These results suggest that *K. pneumoniae* is the most susceptible to the inhibitory effects of MgO-ZnO nanocomposite, requiring the lowest concentration (31.2 μg/mL) to inhibit its growth. In contrast, *S. aureus* and *B. cereus* require a higher concentration (250 μg/mL) to achieve the same effect. For the fungus *C. albicans* (ATCC 10231), the MIC value is also 31.2 μg/mL, which is the same as the most susceptible bacterial strain, *K. pneumoniae.*

In conclusion, the MIC values presented in the table demonstrate the potent antimicrobial activity of MgO-ZnO nanocomposite against both bacterial strains and *C. albicans*. The binary nanoparticles show the highest inhibitory effect on *K. pneumoniae* and *C. albicans*, requiring the lowest concentration (31.2 μg/mL) to inhibit their growth. The synergistic effect of MgO and ZnO, along with their ability to generate ROS, contributes to the antimicrobial properties of these nanoparticles.

In our previous work, we demonstrated that ZnO NPs exhibited exceptional anti-Campylobacter activity, with a MIC 8–16 times lower than that of *E. coli* and *Salmonella* (Xie et al. 2011). The present work did not reveal any statistically significant variations in the inhibitory effects of MgO-ZnO nanocomposite on the growth or inactivation of cells between *C. albicans and K. pneumonia* with the same MIC. However, the difference in MIC values between *C. albicans* and Gram-positive bacteria was threefold lower.

Seghir et al. (2023) demonstrated that mixed oxide nanoparticles exhibit significant antimicrobial activity due to their oxidative stress-inducing properties. For instance, a study noted that the antibacterial action of ZnO NPs is attributed to release of ROS, which can damage the cell membranes and cellular DNA, ultimately leading to cell death. Another study by Anaya-Esparza et al. (2019) emphasized that the production of ROS is a component of the action mechanism that affects membrane fluidity and disrupts cell integrity, corroborating the findings regarding MgO-ZnO nanocomposite.

### Antioxidant activity

**Fig. 8:**
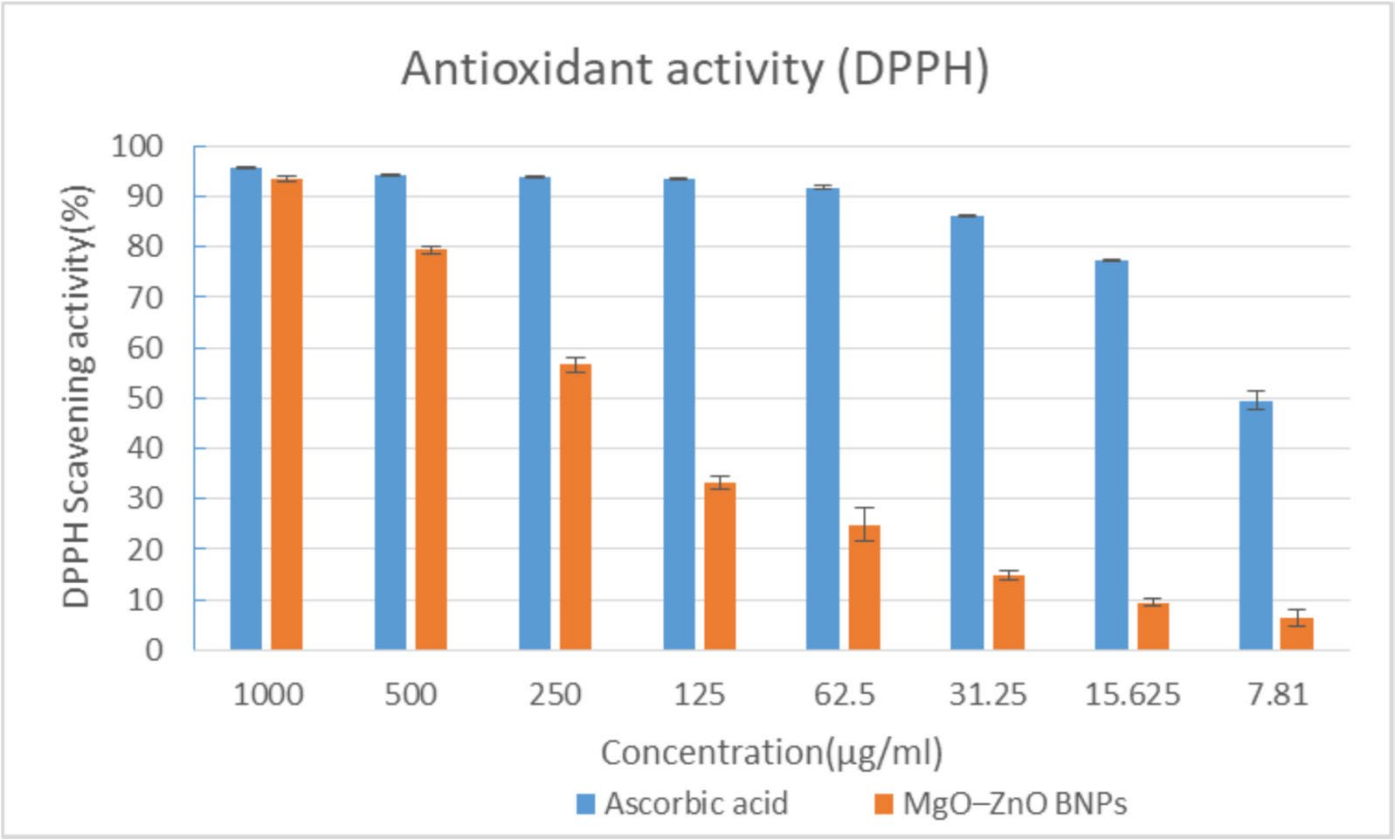
MgO-ZnO nanocomposites' antioxidant activity was measured using DPPH technique at various doses

Much importance has been given to antioxidants since numerous studies proved that a wide range of synthetic and natural substances shows strong antioxidant activity (Lee et al. 2013). In this study, antioxidant activity, which comprises DPPH radical-scavenging activity, has been evaluated using the DPPH technique. The ROS are compounds that in biological mechanisms give rise as a secondary consequence to oppose other molecules (Kurutas 2015). Antioxidants have been used as therapeutic agents because of their anti-atherosclerotic, anti-inflammatory, antiviral, anticancer, anticarcinogenic, and antibacterial qualities (Kalaba et al. 2024). Figure 8 illustrates how the DPPH technique was used in our experiment to assess the antioxidant activity of MgO-ZnO nanocomposite at different doses (1000–7.81 μg/mL). The results demonstrated that MgO-ZnO nanocomposite exhibited superior antioxidant activity with an IC_50_ of 175 μg/mL, compared to ascorbic acid, which had an IC50 of 7.61 μg/mL.

## Conclusion

In conclusion, the innovative green biosynthesis of MgO-ZnO nanocomposite from *P. indica* leaf extract marks a significant advancement in sustainable nanotechnology. This study not only demonstrates the successful production of these nanoparticles but also highlights their favorable biophysical characteristics, ensuring stability within the 5–35 nm size range. The demonstrated anticancer activity, with IC_50_ values of 73.61 µg/mL against Hep-G2 and 31.25 µg/mL against MCF-7, showcases their potential as effective therapeutic agents in cancer treatment. Furthermore, the biosafety profile revealed an IC_50_ of 179.13 µg/mL for the Wi 38 normal cell line, indicating a promising safety margin for further applications. In addition to their anticancer properties, the MgO-ZnO nanocomposite exhibited significant antibacterial activity against various pathogens, including *K. pneumonia* and* E. coli*, along with notable antioxidant effects as determined by the DPPH method. These multifunctional capabilities position these nanoparticles at the forefront of biomedical research, with potential applications in both therapeutic and preventive strategies. This study underscores the importance of exploring plant-mediated synthesis methods, which not only offer a sustainable alternative but also produce nanoparticles with diverse biological activities. Overall, MgO-ZnO nanocomposite presents a promising avenue for future research aimed at developing safe and effective nanomedicine solutions.

## Data Availability

Data supporting the findings of this study are available upon reasonable request.
